# Protective chromosome 1q32 haplotypes mitigate risk for age-related macular degeneration associated with the *CFH-CFHR5* and *ARMS2/HTRA1* loci

**DOI:** 10.1186/s40246-021-00359-8

**Published:** 2021-09-25

**Authors:** Chris M. Pappas, Moussa A. Zouache, Stacie Matthews, Caitlin D. Faust, Jill L. Hageman, Brandi L. Williams, Burt T. Richards, Gregory S. Hageman

**Affiliations:** Steele Center for Translational Medicine, John A. Moran Eye Center, Department of Ophthalmology & Visual Sciences, University of Utah, Salt Lake City, UT 84132 USA

**Keywords:** Age-related macular degeneration, *CFH-CFHR5*, *ARMS2/HTRA1*, Haplotype, Diplotype, Genetic association study

## Abstract

**Background:**

Single-variant associations with age-related macular degeneration (AMD), one of the most prevalent causes of irreversible vision loss worldwide, have been studied extensively. However, because of a lack of refinement of these associations, there remains considerable ambiguity regarding what constitutes genetic risk and/or protection for this disease, and how genetic combinations affect this risk. In this study, we consider the two most common and strongly AMD-associated loci, the *CFH-CFHR5* region on chromosome 1q32 (Chr1 locus) and *ARMS2/HTRA1* gene on chromosome 10q26  (Chr10 locus).

**Results:**

By refining associations within the *CFH-CFHR5* locus, we show that all genetic protection against the development of AMD in this region is described by the combination of the amino acid-altering variant *CFH* I62V (rs800292) and genetic deletion of *CFHR3/1*. Haplotypes based on *CFH* I62V, a *CFHR3/1* deletion tagging SNP and the risk variant *CFH* Y402H are associated with either risk, protection or neutrality for AMD and capture more than 99% of control- and case-associated chromosomes. We find that genetic combinations of *CFH-CFHR5* haplotypes (diplotypes) strongly influence AMD susceptibility and that individuals with risk/protective diplotypes are substantially protected against the development of disease. Finally, we demonstrate that AMD risk in the *ARMS2/HTRA1* locus is also mitigated by combinations of *CFH-CFHR5* haplotypes, with Chr10 risk variants essentially neutralized by protective *CFH*-*CFHR5* haplotypes.

**Conclusions:**

Our study highlights the importance of considering protective *CFH-CFHR5* haplotypes when assessing genetic susceptibility for AMD. It establishes a framework that describes the full spectrum of AMD susceptibility using an optimal set of single-nucleotide polymorphisms with known functional consequences. It also indicates that protective or preventive complement-directed therapies targeting AMD driven by *CFH-CFHR5* risk haplotypes may also be effective when AMD is driven by *ARMS2/HTRA1* risk variants.

**Supplementary Information:**

The online version contains supplementary material available at 10.1186/s40246-021-00359-8.

## Background

Age-related macular degeneration (AMD) is the leading cause of irreversible vision loss in the USA [1, 2] and affects close to 200 million individuals worldwide [3]. Prevalence among individuals over 45 years of age ranges from approximately 7.5% in Asians and Africans to 12.3% among individuals with European ancestry [3]. AMD is characterized by a gradual loss of visual acuity [4, 5], a decrease in contrast sensitivity [6–9] and delays in dark adaptation [10–12], which are associated with progressive photoreceptor loss [13, 14] and impaired retinal pigment epithelium (RPE) metabolism in the macula, the region of the primate eye responsible for high-acuity vision. Patients in the early and intermediate stages of AMD typically present with pigmentary abnormalities, drusen formation and/or pigment epithelium detachments in the fundus. A fraction of patients [3] ultimately progress to the late form of the disease, which is characterized by the gradual atrophy of regions of the retina (geographic atrophy, GA) and/or the abnormal growth of choroidal and/or retinal vessels (neovascular AMD) [15]. Therapeutic options are currently limited to patients with neovascular AMD, although these therapies suffer from inconsistent clinical outcomes [16–21].

Genetic associations with AMD have been extensively studied and are well documented. The most common genetic contributors to AMD are variants associated with a cluster of genes near complement factor H (*CFH*) – complement factor H-related (*CFHR*) 5 on chromosome 1q32 (Chr1 locus) [22–27], and with age-related maculopathy susceptibility 2 (*ARMS2*) and high-temperature requirement factor A1 (*HTRA1*), two tightly-linked genes located on chromosome 10q26 (Chr10 locus) [28, 29]. Genome-wide association studies (GWAS) have identified 32 additional loci associated with AMD, which include *C3*, *C2/CFB* and *CFI*, genes involved in the regulation of the complement system, and genes involved in lipid metabolism and extracellular matrix remodeling. These associations are independent from risk variants on Chr1 and Chr10 [30–33], and only account for a small number of patients with AMD [34–36]. Variants associated with *CFH-CFHR5* and *ARMS2/HTRA1* account for approximately 70% of the variability in AMD explained by additive genetic effects. While they may modulate disease, the other associated loci have a marginal effect when assessing AMD susceptibility [34, 35].

All genetic risk at the Chr10 locus is attributable to the variant rs10490924 (*ARMS2*) or to single-nucleotide polymorphisms (SNPs) in strong linkage disequilibrium (LD) with it [28, 29, 37]. In contrast, multiple SNPs characterize AMD genetic associations within the *CFH-CFHR5* locus [22, 27]. The association between the *CFH* Y402H variant (rs1061170) and increased disease susceptibility was the first to be reported in this region [22, 24, 26]. These early studies found that all common risk haplotypes within the *CFH-CFHR5* region are related to a single risk haplotype with a C allele at *CFH* Y402H [22–24] and that certain haplotypes were associated with a lower risk for AMD [22]. While investigated in multiple studies [22, 25, 27, 30, 38], genetic protection against AMD remains poorly defined, often overlooked [39], and no consensus on causative variants currently exists. This is partly caused by the use of *CFH-CFHR5* risk variants and haplotypes as references when assessing genetic associations with AMD [34, 40, 41] within this locus, which results in the absence of clear distinction between lack of risk and genetic protection. The common missense *CFH* I62 (rs800292) polymorphism is the only amino-acid altering variant that confers protection against AMD [22]. Another form of genetic protection independent of *CFH* Y402H is associated with a common haplotype containing the deletion of *CFHR3* and *CFHR1* (*CFHR3/1* deletion) [25, 38, 42–44]. Haplotypes containing the *CFH* Y402H and *CFH* I62V polymorphisms and the deletion of *CFHR3/1* account for more than 90% of the genetic variability within the *CFH*-*CFHR5* locus [45]. Some of these haplotypes confer an increased risk for AMD, and two of them confer protection against the development of disease. The remaining common haplotypes are present with similar frequencies in cases and controls; they are therefore associated with a lack of risk and referred to as neutral [42, 45–49]. The noncoding variant rs1410996 (or any perfect proxy), which is more strongly associated with AMD than *CFH* Y402H, is also associated with protection against AMD [27, 30, 34, 36]. However, since this variant is shared by protective haplotypes containing the minor allele at *CFH* I62V or the deletion of *CFHR3/1*, the protection associated with it is not entirely independent from that associated with rs800292 or the *CFHR3/1* deletion [33, 42]. To date, true causality between genetic protection and rs1410996, *CFH* I62V or the deletion of *CFHR3/*1 remains to be established [40, 50]. A GWAS performed by the International AMD Genetic Consortium (IAMDGC) defined 8 credible sets of variants within the extended *CFH-CFHR5* region independently associated with AMD [34]. Out of the 8 index SNPs describing the credible sets, one is a proxy for rs1410996 (rs10922109, IAMDGC Locus 1.1, *r*^*2*^ = 0.9919, *D*ʹ = 0.9959), one is a proxy for rs1061170 (rs570618, IAMDGC Locus 1.2, *r*^*2*^ = 0.9914, *D*ʹ = 1) and four (rs121913059, IAMDGC Locus 1.3; rs148553336, IAMDGC Locus 1.4, rs35292876, IAMDGC Locus 1.7 and rs191281603, IAMDGC Locus 1.8) are rare (frequency < 1%). Out of the two remaining common index SNPs, one is associated with risk for AMD (rs187328863, IAMDGC Locus 1.5) while the other (rs61818925, IAMDGC Locus 1.6),  is associated with reduced risk. A haplotype analysis of the extended *CFH-CFHR5* region was recently performed using 7 out of the 8 locus SNPs defined in this GWAS and a SNP tagging the deletion of *CFHR3/1* [41]. The study identified an association between haplotypes based on a proxy for rs1410996 and the index SNP for locus 1.6 and circulating levels of complement factor-related 4 protein. This study and others [51–54] highlight the need to adequately account for genetic protection at Chr1 to fully elucidate the genetic etiology and pathophysiology of AMD.

While many studies have assessed the effect of heterozygosity and homozygosity for *CFH-CFHR5* risk variants on AMD susceptibility [22], very few have considered haplotype combinations (diplotypes) at this locus. In particular, the effect of combinations of *CFH-CFHR5* protective and risk haplotypes on disease susceptibility remains to be determined. Many investigations have established that the effect of *CFH-CFHR5* and *ARMS2/HTRA1* risk variants on AMD susceptibility were independent and additive [28–31, 55, 56]. Individuals homozygous for risk variants at both Chr1 and Chr10 are approximately 32 times more likely to develop AMD as compared to subject with no risk alleles at either locus [56]. So far, no study has considered whether the presence of protective *CFH-CHFR5* haplotypes decrease disease incidence in individuals with risk genotypes at *ARMS2/HTRA1*.

In this study, we first identify the smallest set of variants accounting for common genetic risk and protection against AMD. Analyses of haplotypes and diplotypes based on these SNPs are then performed to assess how protective haplotypes affect AMD susceptibility. We finally characterize the combined effect of Chr1 diplotypes and Chr10 risk variants on AMD risk.

## Results

### Cohort

The case/control cohort included 4787 individuals (Utah: 3306; Iowa: 1481) with a median age of $77.4$ (IQR 12.7; see Table 1). Approximately one-third of the cohort (1587 individuals) consisted of controls. The remaining two-thirds (3200 individuals) presented with AMD in at least one eye. A majority of cases (61.9%) presented with late AMD in at least one eye, with subjects with early or intermediate AMD accounting for 11.7% and 10% of all cases, respectively. The minor allele frequency (MAF) of the variants considered in this study is summarized in Table 2. Frequencies among controls were consistent with frequencies among individuals with European ancestry (EUR) from the 1000 Genomes Project (denoted 1000 G), and among the 17,832 controls used in the IAMGC GWAS [34]. MAF among cases were also consistent with the 16,144 cases from the IAMGC GWAS. The frequency among cases and controls of *CFH-CFHR5* haplotypes based on common IAMDGC index variants (Locus #1.1, #1.2, #1.5, #1.6 with the addition of Locus #1.7) and the deletion of *CFHR3/1* [41] and their associated effect sizes were similar to those of the IAMDGC cohort [41] (see Table 3).

**Table 1:** Characteristics of the case/control cohort

Demographic	Utah	Iowa	Combined cohort
N	3306	1481	4787
Age, median (IQR)	75.8 (13.2)	79.9 (11.1)	77.4 (12.7)
Males	1243	551	1794
Females	2057	930	2987
*Controls*			
N	1228	359	1587
Age, median (IQR)	71.6 (10.5)	77.8 (12.6)	72.8 (11.6)
Males	473	169	642
Females	755	190	945
*Cases*			
N	2078	1122	3200
Age, median (IQR)	78.8 (12.1)	80.4 (10.3)	79.45 (11.6)
Males	770	382	1152
Females	1302	740	2042
Early AMD	445	245	690 (11.7%)
Intermediate AMD	443	85	528 (10%)
Late AMD (atropy)	251	83	334 (10.4%)
Late AMD (neovascular)	852	666	1518 (47.4%)
Late AMD (atrophy and neovascular)	87	43	130 (4.1%)
Late AMD (combined)	1190	792	1982 (61.9%)

**Table 2:** Frequencies of *CFH-CFHR5* and *ARMS2/HTRA1* variants associated with AMD among individuals from the 1000 Genomes Project phase 3 (1000 G) and controls and cases, with associated effect sizes and *p*-values

Variant (Position) Major/minor allele	Minor allele frequency (MAF)			OR (95% CI)	*p*-value*	IAMDGC (17,832 controls, 16,144 cases) [41]			
						MAF		OR	*p*-value
	1000 G	Controls	Cases			Controls	Cases		
**IAMGC Locus # 1.5** rs187328863 (chr1:196380158) C/T (+)	0.028	0.029	0.053	2.12 [1.63; 2.74]	1.29e−08	0.028	0.054	2.27	1.1e−68
***CFH* I62V** rs800292 (chr1:196642233) G/A (−)	0.260	0.241	0.142	0.52 [0.46; 0.58]	1.83e−29	n.a	n.a	0.49^(1)^	7.94e−286
***CFH* Y402H** **IAMGC Locus # 1.2** rs1061170 (chr1:196659237) T/C (+)	0.362	0.369	0.559	2.27 [2.06; 2.49]	1.16e−64	0.37^(2)^	0.58^(2)^	2.38^(2)^	2.0e−590^(2)^
**IAMGC Locus # 1.1** rs1410996 (chr1:196696933) G/A (−)	0.425	0.418	0.237	0.41 [0.37; 0.45]	1.04e−66	0.43^(3)^	0.22^(3)^	0.38^(3)^	9.6e−618^(3)^
***CFHR3/1* Deletion** rs12144939 (chr1:196698945) G/T (−)	0.190^(4)^	0.199	0.108	0.45 [0.39; 0.51]	1.43e−34	0.21^(4)^	0.11^(4)^	0.48^(4)^	2.19e−273^(4)^
**IAMGC Locus # 1.6** rs61818925 (chr1:196815450) G/T (−)	0.422	0.340	0.256	0.65 [0.59; 0.72]	1.68e−17	0.385	0.284	0.60	6.0e−165
***ARMS2/HTRA1*** **IAMGC Locus # 17** rs10490924 (chr10:124214448) G/T (+)	0.195	0.215	0.381	2.33 [2.10; 2.59]	1.59e−56	0.208^(5)^	0.436^(5)^	2.81^(5)^	6.5e−735^(5)^

Frequencies and effect size from the IAMDGC study are also provided

^(1)^Estimated using the MAF in the 1000 G project (see Methods). ^(2)^Based on the perfect proxy rs570618 (*r*^*2*^ = 0.9914, *D*ʹ = 1.0). ^(3)^Based on the perfect proxy rs10922109 (*r*^*2*^ = 0.9919, *D*ʹ = 0.9959). ^(4)^Based on rs6677604, which is another tag for the *CFHR3/1* deletion with minor allele A. ^(5)^Based on the perfect proxy rs3750846 (*r*^*2*^ = 1.0, *D*ʹ = 1.0). *Bonferroni correction for multiple testing of 7 variants = 0.007 (0.05/7)

**Table 3:**
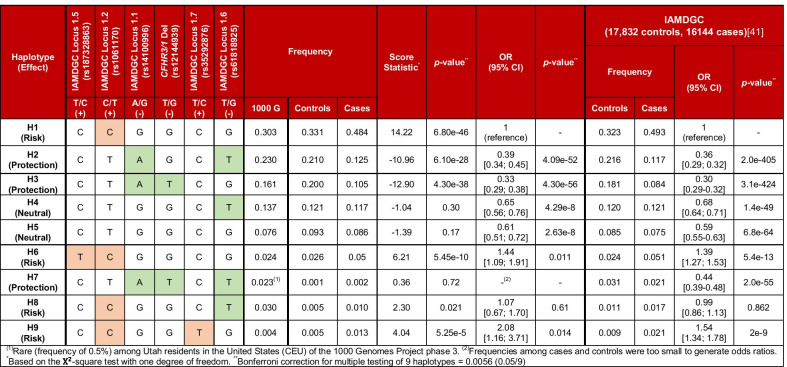
Haplotype analysis of the *CFH-CFHR5* extended region in the combined Utah/Iowa cohort using all common credible sets of variants independently associated with AMD (IAMDGC Locus 1.1, 1.2, 1.5 and 1.6) and comparison with the IAMDGC cohort (17,832 controls, 16,144 cases). Frequencies among Caucasians from the 1000 Genomes Project phase 3 (EUR), denoted 1000 G, are also provided

The labelling and numbering of haplotypes follows that of the haplotype analysis of the IAMDGC cohort [41]. For consistency with this analysis, the rare variant rs35292876 (IAMDGC Locus # 1.7 with minor allele T, MAF_controls_ = 0.005; MAF_cases_ = 0.014; OR 2.99 [1.73; 5.17], *p* = 8.8e−5 in our cohort and MAF_controls_ = 0.009; MAF_cases_: 0.021; OR 2.42, *p* = 8.2e−37 in the IAMDGC study) was also included. The rs35292876 minor allele exists exclusively on a low-frequency haplotype containing a C (risk) allele at rs1061170. While this variant may modulate risk, its frequency and effect size are therefore accounted for by haplotypes with a C allele at rs1061170. Haplotypes in the IAMDGC cohort used rs570618 in place of rs1061170 (*r*^*2*^ = 0.9914, *D*ʹ = 1.0), rs10922109 in place of rs14100996 (*r*^*2*^ = 0.9919, *D*ʹ = 0.9959) and rs6677604 in place of rs12144939

^(1)^Rare (frequency of 0.5%) among Utah residents in the USA (CEU) of the 1000 Genomes Project phase 3. ^(2)^Frequencies among cases and controls were too small to generate odds ratios *Based on the ${\mathrm{\rm X}}^{2}$-square test with one degree of freedom. **Bonferroni correction for multiple testing of 9 haplotypes = 0.0056 (0.05/9)

### Protection at the *CFH-CFHR5* locus is explained entirely by the combination of *CFH* I62V and *CFHR3/1* deletion

**Fig. 1:**
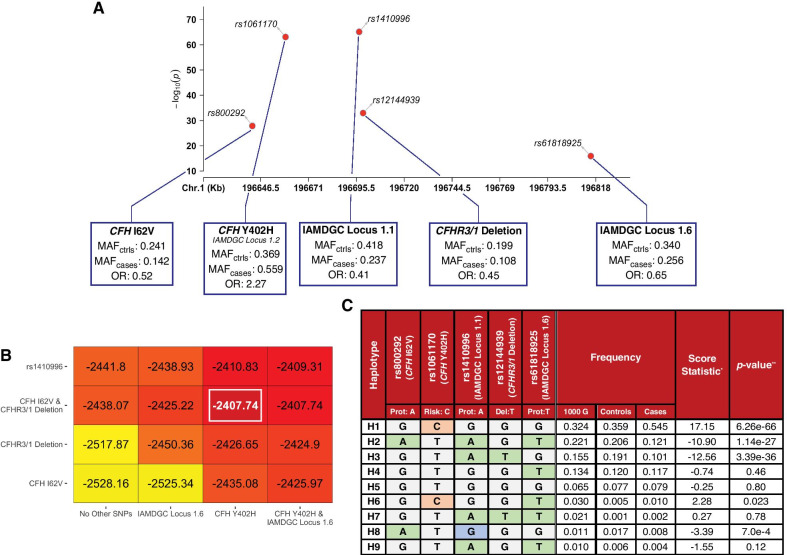
Common protection at the *CFH-CFHR5* locus is entirely described by the combination of *CFH* I62V and the deletion of *CFHR3/1*. **a** Manhattan plot showing the four common variants associated with protection against AMD (*CFH* I62V, rs1410996, the *CFHR3/1*-tagging rs12144939 and IAMDGC Locus 1.6) and the risk-conferring *CFH* Y402H. **b** Heatmap of the log-likelihood of additive regression models conditioning variables on the vertical axis to those of the horizontal axis. The combination of *CFH* I62V, the *CFHR3/1* deletion and *CFH* Y402H yields the best model (boxed and highlighted). **c** Haplotype analysis using the four common variants associated with protection against AMD and *CFH* Y402H. Frequency for rs12144939 among Caucasians from the 1000 Genomes Project was based on rs6677604. *Score statistic based on the ${\rm X}^{2}$ test statistic with 1 degree of freedom. **Bonferroni correction for multiple testing of 9 haplotypes = 0.0056 (0.05/9)

Genetic protection within the *CFH-CFHR5* extended region is generally accounted for by the noncoding variant rs1410996 (or any proxy) [27, 30, 34, 36], which is the SNP most strongly associated with AMD protection at this locus (OR 0.41 [0.37; 0.45], *p* = 1.04e−66 in our cohort); see Fig. 1a. The *CFH* I62V polymorphism (rs800292, OR: 0.52 [0.46; 0.58], *p* = 1.83e−29), the SNP tagging the deletion of *CFHR3/1* (rs12144939, OR: 0.45 [0.39; 0.51], *p* = 1.43e−34) and the index SNP for the IAMDGC Locus 1.6 (rs61818925, OR: 0.65 [0.59; 0.72], *p* = 1.68e−17) are also associated with protection, but with comparatively larger odd ratios. Out of these four SNPs, we sought to identify the set of variants that accounts for all common genetic protection against AMD. To do so, we compared regression models including additive combinations of rs800292, rs12144939, rs1410996 and rs61818925 while controlling for age and sex using log-likelihood tests (see Fig. 1b). We found that the model including the two variants rs800292 and rs12144939 only was a significantly better fit than the model including rs1410996 only (*p* = 0.0063). When conditioning on *CFH Y402H*, the model including rs800292 and rs12144939 only was superior to the one including rs1410996 only (*p* = 0.013), which was consistent with a previous observation [50]. Adding rs61818925 to the model including rs800292, rs12144939 and *CFH* Y402H did not significantly increase the log-likelihood (*p* = 0.93).

To elucidate why *CFH* Y402H, *CFH* I62V and the deletion of *CFHR3/1* are a superior set of variants to describe common risk and protection against AMD, we performed a haplotype analysis of the *CFH-CFHR5* locus using rs800892, rs1061170, rs140996, rs12144939 and rs61818925 (see Fig. 1c). We identified three common protective haplotypes (H2, H3 and H8) with a frequency > 1% in our cohort. All of these haplotypes carry a protective allele (A) at rs800292 (H2, H8) or have the *CFHR3/1* deletion (T allele at rs12144939, H3). Nearly all (98.4%) rs1410996 chromosomes with the protective A allele contain either the protective allele at *CFH* I62V or the deletion of *CFHR3/1*. This variant (and any SNP in LD with it) is therefore a proxy for the combination of *CFH* I62V and the deletion of *CFHR3/1*. One haplotype (H5), with frequency of 1.9% among our controls and 1% among cases, does not carry the protective allele at rs1410996 despite having a protective allele at *CFH* I62V (see Additional file 1: Table S2). In addition, one haplotype (H7) with frequency > 1% among individuals with European ancestry of the 1000 Genomes Project (but rare among our cases and controls) was not associated with protection against AMD despite carrying the minor allele at rs1410996 (*p* = 0.10). The protective allele at rs61818925 (T) is only part of one protective haplotype (H2), which contains the A allele at *CFH* I62V. The T allele at rs61818925 is also part of a haplotype with a C allele at *CFH* Y402H (H6) that is twice as frequent among our cases than in our controls, and of haplotypes with no significant association with AMD (H4 and H9). The presence of this allele on risk, neutral and protective haplotypes, in addition to the fact that it provides no additional information to our regression models, led us to exclude rs61818925 from further analyses.

### Haplotypes based on *CFH* I62V, *CFHR3/1* deletion and *CFH* Y402H differentiate disease susceptibility at the *CFH-CFHR5* locus

The risk allele (T) for the IAMDGC Locus 1.5 (rs187328863 OR: 2.12 [1.63; 2.74], *p* = 1.29e−8), which was independently associated with increased risk for AMD in a previous GWAS [34], exists exclusively on a low-frequency haplotype containing a C (risk) allele at rs1061170 (see Table 3). While this variant may modulate risk, its frequency and effect size suggest that this risk is generally accounted for by haplotypes with a C allele at rs1061170.

The combination of rs800292, rs1061170 and rs12144939 yields four common haplotypes, which capture 99% of control- and 99.5% of case-associated chromosomes in our cohort (see Table 4 and Additional file 1: Table S3). Compared to a haplotype analysis of the extended *CFH-CFHR5* region using 7 out of the 8 IAMDGC Locus SNPs (including four rare variants) and a SNP tagging the deletion of *CFHR3/1* [41], we can estimate that haplotypes based on these 4 variants comprise at least 96.7% of control- and 92.8% of case-associated chromosomes of the IAMDGC cohort. A common haplotype (H3) has a frequency similar (approximately 20%) among cases and controls (${\chi }^{2}=-1.31,$ *p* = 0.19) and is therefore neutral against AMD. Because this haplotype describes the absence of genetic risk or protection for developing AMD, it was used as a reference to describe the full spectrum of AMD susceptibility in place of the most common haplotype. The most common haplotype (H1) is associated with an increased risk for AMD when compared to H3 and carries a C allele at rs1061170 (OR = 1.61 [1.42; 1.83], *p* = 1.2e−13). Two common protective haplotypes carry either a protective allele at *CFH* I62V (referred to as Prot-I62, OR = 0.61 [0.52; 0.71], *p* = 1.9e−10) or the deletion of *CFHR3/1* (referred to as Prot-Del, OR = 0.53 [0.45; 0.62], *p* = 3.9e−14). A fifth haplotype (H5) with frequency of 1.1% in the 1000 Genomes Project carries both a protective allele at *CFH* I62V and the *CFHR3/1* deletion. While rare in our cohort, this haplotype is twice as frequent in our controls than in our cases (See Additional file 1: Table S3).

**Table 4:**
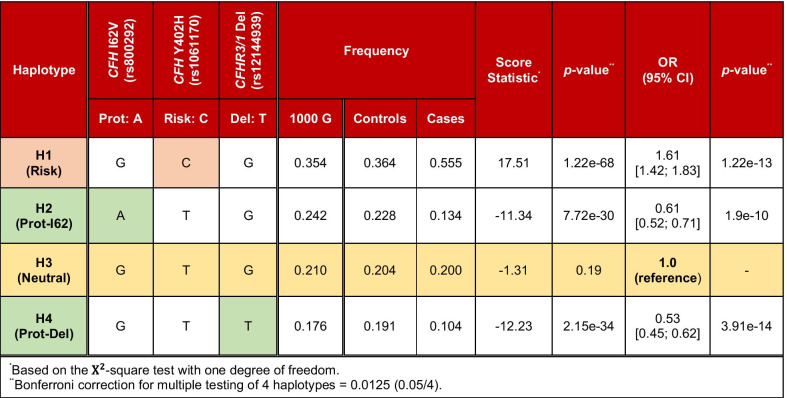
Haplotypes based on the protection-conferring *CFHR3/1* deletion, *CFH* I62V and the risk variant *CFH* Y402H

The common neutral haplotype H3, which describes the absence of genetic risk or protection for developing disease, was used as the reference haplotype to describe the full spectrum of AMD susceptibility at the *CFH-CFHR5* locus

^*^Based on the ${\mathrm{\rm X}}^{2}$-square test with one degree of freedom

^**^Bonferroni correction for multiple testing of 4 haplotypes = 0.0125 (0.05/4)

### Genetic risk at the *CFH-CFHR5* locus is determined by combinations of protective and risk haplotypes on Chr1

**Fig. 2:**
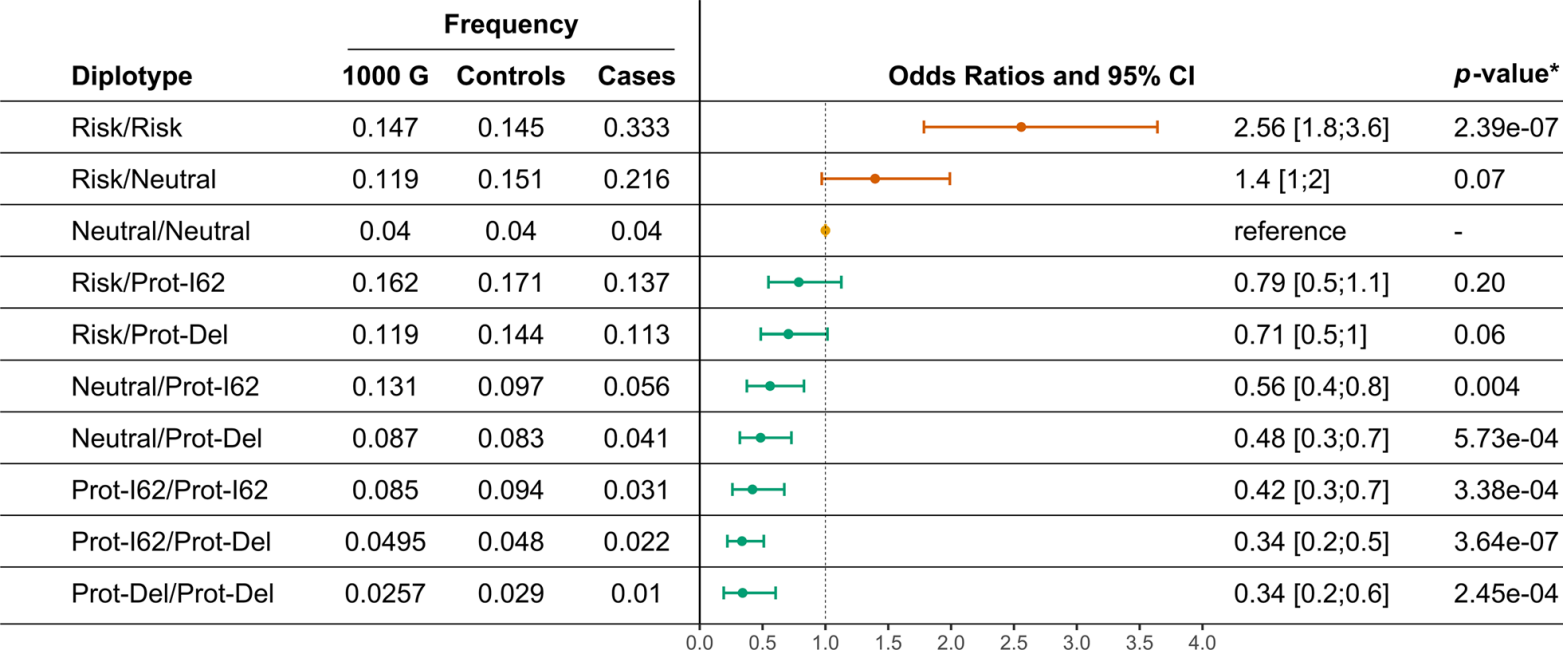
Association between haplotypes combinations (diplotypes) based on the protection conferring *CFHR3/1* deletion and *CFH* I62V and the risk variant *CFH* Y402H. The common neutral diplotype Neutral/Neutral, which describes the absence of genetic risk or protection, was used as the reference diplotype to describe the full spectrum of AMD susceptibility at the *CFH-CFHR5* locus. *Bonferroni correction for multiple testing of 10 diplotypes = 0.005 (0.05/10)

Ten *CFH-CFHR5* haplotype combinations (diplotypes) with frequencies higher than 1% were present among our cases and controls (see Fig. 2 and Additional file 1: Table S4). The frequency of combinations of neutral haplotypes (Neutral/Neutral diplotype) did not differ significantly between cases and controls (frequency of 4%, ${\chi }^{2}=0.06,$ *p* = 0.80). This diplotype is therefore neutral and was used as a reference to differentiate disease susceptibility in our cohort. Overall, we found that combinations of *CFH-CFHR5* haplotypes strongly influence AMD susceptibility. Risk/Risk (OR: 2.56 [1.8; 3.6]) and Risk/Neutral (OR: 1.4 [1.0; 2.0]) diplotypes confer an increased risk for AMD. Notably, individuals with combinations of risk and protective haplotypes are generally protected against AMD, with odds ratios ranging from 0.71 (CI [0.5; 1.0]) for Risk/Prot-Del diplotypes to 0.79 (CI [0.5; 1.1]) for Risk/Prot-I62 diplotypes. The strongest form of genetic protection is found among individuals homozygous for the Prot-Del haplotype (OR: 0.34 [0.2; 0.6]).

### Risk and protective *CFH-CFHR5* haplotypes strongly influence risk at the *ARMS2/HTRA1* locus

**Fig. 3:**
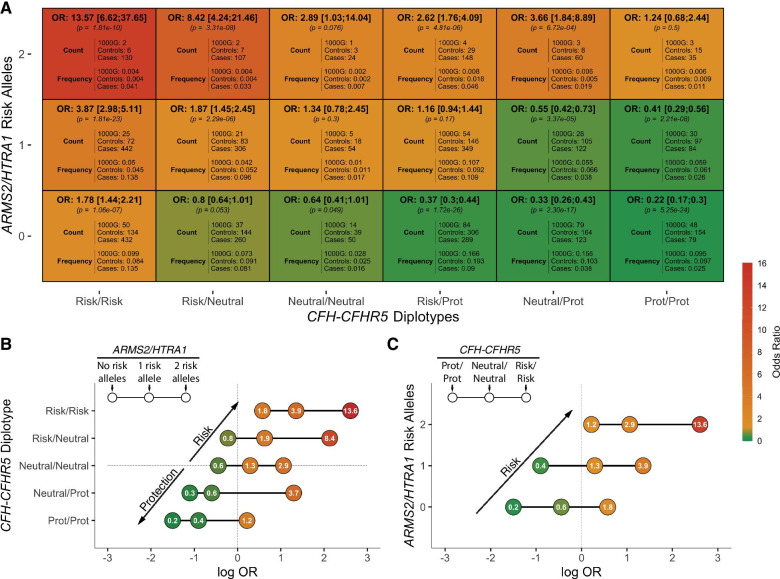
Association between *CFH-CFHR5* and *ARMS2/HTRA1* diplotype combinations and AMD susceptibility. The effect size was calculated using Firth’s bias-reduced logistic regression [57] while adjusting for age and gender, and were used to define the colormap in all sub-figures. Bonferroni correction for multiple testing of 18 diplotype combinations = 0.0028 (0.05/18). **a** Odds ratios and frequency among cases and controls for *CFH-CFHR5* and *ARMS2/HTRA1* diplotype combinations. **b** Effect of *CFH-CFHR5* diplotypes on AMD susceptibility among individuals with zero (left circle), one (middle circle) or two (right circle) *ARMS2/HTRA1* risk alleles and associated odds ratios (provided in circles). AMD susceptibility moves towards protection with protective haplotypes on Chr1. **c** Effect of *ARMS2/HTRA1* risk alleles on AMD susceptibility among individuals with Prot/Prot (left circle), Neutral/Neutral (middle circle) or Risk/Risk (right circle) *CFH-CFHR5* diplotypes and associated odds ratios (in circles)

Since protective *CFH-CFHR5* haplotypes strongly influence AMD susceptibility on Chr1, we sought to determine if they had any effect on risk associated with the second most common and strongly AMD-associated locus, the *ARMS2/HTRA1* gene. Risk at this locus is tagged by the variant rs10490924 (OR 2.33 [2.10; 2.59], *p* = 1.59e−56 in our cohort). Consistent with previous reports [28–31, 55, 56], there is no evidence of epistasis between *CFH-CFHR5* and *ARMS2/HTRA1*, and the contribution of risk and protective *CFH-CFHR5* haplotypes to Chr10 risk is additive in nature (see Fig. 3a and Additional file 1: Fig. S1). In the absence of risk or protective haplotypes on Chr1 (Neutral/Neutral diplotype), odds ratios in our case control cohort range from 0.64 (95% CI [0.41; 1.01], *p* = 0.049) among individuals with no *ARMS2/HTRA1* risk alleles to 1.34 (95% CI [0.78; 2.45], *p* = 0.076) when one *ARMS2/HTRA1* risk allele is present and 2.89 (95% CI [1.03; 14.04], *p* = 0.076) among subjects with two *ARMS2/HTRA1* risk alleles.

Overall, AMD susceptibility among individuals with 0, 1 or 2 *ARMS2/HTRA1* risk alleles moves towards protection when protective haplotypes are present on Chr1 (see Fig. 3b). When compared to individuals with two *CFH-CFHR5* Neutral/Neutral diplotypes, the presence of two protective haplotypes (Prot-I62 or Prot-Del, combined in Fig. 3) reduces odds ratios 2.3-fold (OR reduced from 2.89 to 1.24; two *ARMS2/HTRA1* risk alleles) or 3.3-fold (OR reduced from 1.34 to 0.41; one *ARMS2/HTRA1* risk allele). When considering the effect size associated with combinations of risk, neutral and protective haplotypes on Chr1 and risk alleles on Chr10, we find that, as expected for additive genetic contributions, *ARMS2/HTRA1* risk alleles are counteracted by protective *CFH*-*CFHR5* haplotypes in an approximately one-to-one manner (see Fig. 3b and Additional file 1: Fig. S2). At the other end of the AMD susceptibility spectrum, the presence of two *CFH*-*CFHR5* risk haplotypes increases odds ratios associated with the Neutral/Neutral diplotype 2.8-fold (OR increased from 0.64 to 1.78; no *ARMS2/HTRA1* risk alleles) to 4.7-fold (OR increased from 2.89 to 13.57; two *ARMS2/HTRA1* risk alleles). In comparison, AMD susceptibility only moves towards risk with *ARMS2/HTRA1* risk alleles, regardless of diplotypes on Chr1 (see Fig. 3c). This is because the spectrum of susceptibility associated with this locus only ranges from lack of risk (no risk alleles) to risk (one or two risk alleles).

## Discussion

Genetic protection within the 1q32 *CFH-CFHR5* extended region is generally accounted for by the noncoding variant rs1410996 (IAMDGC Locus 1.1). This variant tags two independent protective haplotypes that include the *CFH* I62 allele or genetic deletion of *CFHR3/1*, but rarely both [40, 42, 50]. It is likely that rs1410996 was identified as the most likely causal variant by the IAMDGC GWAS (for signal 1.1) [34] and as an independent AMD-associated variant by others [25, 30] precisely because of these combined haplotype effects. To demonstrate this, we can apply the same methodology as the one used by the IAMDGC GWAS authors to illustrate a counterexample of credible set variants able to depict the most likely causal variants in the presence of haplotype effects (Supplementary Figure S4 of the original publication [34]). Our haplotype analysis (Additional file 1: Table S2) indicates that one protective haplotype, with a frequency of 1.9% among controls and 1% among cases, carries the protective allele at *CFH* I62V but not at rs1410996. In addition, one haplotype with frequency > 1% among individuals with European ancestry of the 1000 Genomes Project (but rare among our cases and controls) is not associated with protection against AMD despite carrying the minor allele at rs1410996 (*p* = 0.10). This suggests that it is not the A allele of rs1410996 that carries protection but rather its coinciding with the protective allele of *CFH* I62V and the *CFHR3/1* deletion. Our analysis therefore indicates that causality for protection at the *CFH-CFHR5* locus is more likely to originate from *CFH* I62 or the deletion of *CFHR3/1* than from rs1410996. This idea is further supported by the fact that unlike rs1410996 or variants in LD with it, both of these variants are protein altering. The minimal number of *CFH-CFHR5* SNPs used to define risk, neutrality and protection associated with Chr1 could effectively be reduced to rs1061170 and rs1410996 without losing many chromosomes. However, these two variants are not sufficient to identify the origin of genetic protection, which is essential to elucidate the pathophysiology of AMD and identify viable therapeutic targets.

While being associated with a lower risk for AMD [34], rs61818925 (IAMDGC Locus 1.6) shows no added protection against AMD and does not explain any risk or protection that could not be attributed to *CFH* 402H, *CFH* I62 or the *CFHR3/1* deletion. It is likely that the significance of the association between rs61818925 and AMD results from its partial LD with *CFH* I62V (*r*^*2*^ = 0.29, *D*ʹ = 0.78). Our results are consistent with a published haplotype analysis performed using the IAMDGC cohort [41]. In this study, IAMDGC Locus 1.6 showed no added protection in H4 (vs H5), H7 (vs H3) or H8 (vs H1) (see Table 3). The only other haplotype containing the minor allele at rs61818925 is H2. This haplotype contains the minor allele at rs1410996 without the *CFHR3/1* deletion and therefore predominantly contains the protective allele at rs800292. In agreement with a previous analyses [41], we found that risk associated with rs187328863 (IAMDGC Locus 1.5) is accounted for by haplotypes with a C allele at rs1061170 (see Table 3). It is unclear if this intronic variant, which is located within the *KCNT2* gene, has any functional consequences.

Narrowing the number of variants necessary to define genetic susceptibility at *CFH-CFHR5* allows for the analysis of diplotypes and the assessment of AMD susceptibility in attainable sample sizes. Considering *CFH-CFHR5* diplotypes is a robust and accurate way of assessing AMD susceptibility at this locus. For instance, whereas Risk/Neutral and Risk/Prot diplotypes are both associated with a C/T genotype when considering rs1061170 only, our study shows that these two combinations are in fact associated with very distinct susceptibilities for AMD (risk and protection, respectively). The concept of risk, neutrality and protection, which has been used in other studies [42, 45–49], provides an intuitive framework to understand how variants affect AMD outcomes. Using the risk haplotype H1 as a reference [34, 40, 41] obscures the fact that some haplotypes are present with similar frequencies among cases and controls, and are therefore not associated with AMD. Conversely, the use of neutral haplotypes as a reference simplifies the identification of risk haplotypes, which are more common in cases, and protective haplotypes, which are more common in controls. Unlike most genetic disease-associated loci where risk and protection are binary, lack of risk at *CFH-CFHR5* does not imply protection and vice-versa. This is especially important in light of our finding that Chr1 protective haplotypes lower risk originating from the presence of one or two risk alleles at the *ARMS2/HTRA1* locus, the other major driver of AMD.

Our study demonstrates that *CFH* I62V, *CFH* Y402H and a *CFHR3/1* deletion tagging-SNP form the smallest set of variants necessary to fully differentiate the most common AMD susceptibility associated with the *CFH-CFHR5* extended region. It also suggests that associations of common variants within this locus [22–24, 26–30, 34, 36, 37] can be traced back to the protein function altering changes at position 62 and 402 affecting the CFH protein and its splice variant, factor H-like protein 1 (FHL-1), and to the loss of the FHR-1 and FHR-3 proteins. The CFH/FHL-1 H402 allotype has been shown to alter the binding specificity of the CFH protein at the interface between the retinal pigment epithelium and Bruch’s membrane [58–61], which is the relevant location of AMD pathology, and for glycosaminoglycans [62, 63]. The CFH and FHL-1 I62 allotype is associated with increased complement co-factor activity, which may result in reduced complement activation and protection against AMD [46, 53]. Several studies have shown that FHR-1 and FHR-3 proteins compete with CFH and FHL-1 for binding to C3b and other ligands [42, 64]. Their loss is associated with an enhanced regulation by CFH/FHL-1 that leads to protection against AMD. A recent study reported that lower circulating levels of complement factor-related 4 (FHR-4) protein were associated with a lower risk for AMD [41]. Previous work showed that the protective rs1410996 allele had the strongest association with reduced FHR-4 levels within the *CFH-CFHR5* region and that this association was independent from the loss of the FHR-1 and FHR-3 [65]. Our results indicate that the association between lower FHR-4 levels and reduced AMD risk is likely driven by *CFH* I62V or SNPs in LD with it, although more work is necessary to confirm this. These studies and others [41, 51] highlight the importance of considering the effect of *CFH* I62V, *CFH* Y402H and *CFHR3/1* deletion in genotype/phenotype association studies.

The mechanisms associated with AMD driven by *ARMS2/HTRA1* risk variants have yet to be elucidated. The AMD-associated region within this locus has recently been narrowed to a block of SNPs overlapping *ARMS2* exon 1 and intron 1 [37]. Due to conflicting reports, it is not yet clear if the Arms2 protein is present in human tissue and cells [66–69]. The HtrA1 protein functions as both a secreted serine protease and an extracellular chaperone [70], and cleaves a variety of extracellular matrix (ECM) proteins, proteoglycans and growth factors [71, 72]. Evidence suggests that the biological and disease initiation events associated with AMD driven by risk at Chr10 are distinct from Chr1-directed AMD [51, 73]. However, the observed mitigating effect of protective *CFH-CFHR5* on *ARMS2/HTRA1* risk indicates that therapeutic interventions targeting the complement system may potentially modulate risk on Chr10. We did not have the power to investigate the effect of protective *CFH-CFHR5* haplotypes on risk associated with loci other than *ARMS2/HTRA1*, but by showing that protection in the *CFH-CFHR5* region alleviates risk in the two loci responsible for the majority of AMD, our study does indicate that targeting the complement system may have beneficial effects even when AMD is driven by other loci.

## Conclusions

Our study demonstrates that all associations between common *CFH-CFHR5* variants and AMD reported to date can be explained by the variants that alter CFH protein function (*CFH* I62V and *CFH* Y402H) and by the genetic deletion of *CFHR3/1*. It also shows that genetic susceptibility to AMD associated with the *CFH*-*CFHR5* and *ARMS2*/*HTRA1* loci is mitigated by protective *CFH-CFHR5* haplotypes. These protective haplotypes counteract *CFH-CFHR5* risk haplotypes significantly, so much so that individuals with risk/protective haplotype combinations are generally protected against the development of AMD. They also essentially neutralize the effect of *ARMS2/HTRA1* risk polymorphisms, which indicates that protective complement-directed therapies designed to prevent AMD driven by *CFH-CFHR5* risk haplotypes may also be effective when AMD is driven by *ARMS2/HTRA1* risk variants.

## Methods and materials

### Cohort

Subjects were recruited between 2009 and 2019 at the Steele Center for Translational Medicine, John A. Moran Eye Center, University of Utah, USA, and between 1999 and 2009 at the University of Iowa, Iowa City, Iowa, USA, as part of a case/control study of the genetic etiology of AMD. The study adhered to the tenets of the Declaration of Helsinki and was approved by the Institutional Review Boards of the University of Utah and University of Iowa. All participants provided informed written research consent at the two locations. All subjects were Caucasian, unrelated and older than 55. Venous blood and/or saliva and demographic data including age, gender, ethnicity, smoking history were collected at the time of recruitment.

### Grading

For each subject, both eyes were graded by the same two independent experienced observers based on fundus photographs and/or spectral domain optical coherence tomography volume scans collected at the time of recruitment. Grading was based on the international classification of mutually exclusive stages of age-related maculopathy introduced by the Rotterdam Group [74] and is detailed in Additional file 1: Table S1. Patients with no clinically observable signs of AMD were classified as controls (grade 0). Patients were classified as cases by the presence of drusen less than 63 µm in diameter, soft distinct drusen (≥ 63 µm in diameter) with or without pigmentary changes, isolated pigmentary changes without drusen (≥ 63 µm in diameter), soft indistinct drusen (≥ 125 µm in diameter) with or without pigmentary changes, geographic atrophy and/or neovascular AMD.

### SNP selection

We selected SNPs within the *CFH-CFHR5* and *ARMS2/HTRA1* regions on the basis of previous genetic association analyses [22–24, 26–30, 37] including genome wide association studies [34, 36]. We considered SNPs with minor allele frequency > 1% and set a minimum threshold value of 0.8 for the *r*^*2*^ linkage disequilibrium parameter. Therefore, the SNPs selected accounted for all common variants associated with AMD within the *CFH-CFHR5* and *ARMS2/HTRA1* regions. Three SNPs, including rs10490924, were genotyped within the *ARMS2/HTRA1* region in all samples. Thirty-seven SNPs were genotyped in the extended *CFH-CFHR5* region; they included *CFH* I62V (rs800292), *CFH* Y402H (rs1061170), rs1410996 and the *CFHR3/1* deletion tagging SNP rs12144939. Copy number variant assays were performed to validate the use of rs12144939 as a tagging SNP for the *CFHR3/1* deletion. Common (frequency > 1%) index variants identified by Fritsche [34] that were not in LD (*r*^*2*^ > 0.8) with these SNPs were also genotyped. These included rs187328863 (IAMGC Locus 1.5) and rs61818925 (IAMDGC Locus 1.6).

### Genotyping

Genomic DNA was isolated from peripheral blood leukocytes with QIAamp DNA Blood Maxi kits (Qiagen, Valencia, CA). Genotyping was performed by TaqMan assays (Applied Biosystems, Foster City, California) using 10 ng of template DNA in a 5µL reaction. When available, pre-designed assays were used. When pre-designed assays were unavailable, custom assays were designed using the manufacturer’s design software. The thermal cycling conditions in the 384-well thermocycler (PTC-225, MJ Research) consisted of an initial hold at 95 °C for 10 min, followed by 40 cycles of a 15-s 95 °C denaturation step and a 1-min 60 °C annealing and extension step. Plates were read in the 7900HT Fast Real-Time PCR System (Applied Biosystems).

### Quality control

Data cleaning and quality control checks were performed using PLINK (v1.9) [75]. Heterogeneity between subjects recruited in Utah and Iowa was assessed using Cochran’s Q-statistic [76] and the *I*^*2*^ metric [77] while adjusting for age and sex. We found no evidence of heterogeneity when considering SNPs and haplotypes in the *CFH-CFHR5* region (${I}^{2}=0\%, p > 0.47$). Low heterogeneity was detected when considering the rs10490924 (${I}^{2}=42\%$) with a non-significant Q-statistic ($p=0.19$). The Utah and Iowa cohorts were therefore combined without resorting to meta-analyses approaches. Linkage disequilibrium analyses were performed using the R package LDlinkR [78] and populations of Caucasian descent from the 1000 Genomes Project phase 3 [79].

### Allele frequencies

Minor allele frequencies were generated using PLINK (v1.9) [75]. Allele frequencies among Caucasians were obtained from the 1000 Genomes Project phase 3 [79] (https://www.ncbi.nlm.nih.gov/variation/tools/1000genomes/) and the R [80] package *LDlinkR* [78]. Estimates were obtained by combining Utah residents in the USA (CEU), residents from Toscani in Italy (TSI), Finnish individuals in Finland (FIN), British individuals in England and Scotland (GBR) and subjects from the Iberian population in Spain (IBS). When available, frequencies among cases and controls of the International AMD Genetic Consortium (IAMDGC) were collected from publicly available sources [34, 41]. In the 1000 Genomes Project phase 3 and IAMDGC study, the frequency of the *CFHR3/1* deletion was determined based on rs6677604 (minor allele A).

### Haplotype phasing and analyses

Haplotype analyses were performed in R [80] using the package *haplo.stats* [81]. The package uses an EM algorithm to analyze indirectly measured haplotypes and assumes that all subjects are unrelated. The *haplo.glm* function was used to perform generalized regressions of AMD status on haplotype effects. The function uses the posterior probabilities of pairs of haplotypes per subjects as weights to update regression coefficients. Phasing was generated for our cohort and for Caucasians from the 1000 Genomes Project phase 3 [79] (genotype data for the SNPs of interest were downloaded from https://www.ncbi.nlm.nih.gov/variation/tools/1000genomes/) using the same R package.

### Association analyses

Association analyses were performed using PLINK (v1.9) [75] and R [80]. Associations between AMD single variants, haplotypes and diplotypes were assessed using the ${\chi }^{2}$ test for association and logistic regressions under additive models including sex and age as covariates. Epistasis between genes was assessed by including a multiplicative interaction term in the logistic regression models. Odds ratios and confidence intervals for *CFH-CFHR5* and *ARMS2/HTRA1* diplotype combinations were estimated by applying Firth’s bias-reduced logistic regression [57] using the R package *brglm2* [82]. Multiple testing was accounted for by adjusting the significance level using Bonferroni corrections. Manhattan plots were generated using the R package *CMplot* [83]. When considering multiple variants, the most parsimonious best-fit regression was determined on the basis of likelihood ratio test statistics.

### Comparison with IAMDGC GWAS

Frequencies and effect sizes of variants and haplotypes of interest were compared to those of the GWAS published in 2016 by the International Age-related Macular Degeneration Genomics Consortium (IAMDGC) [34]. The study used 16,144 cases and 17,832 controls of European descent. The publicly available summary statistics include genotyped SNPs, *p*-values and directions of associations. Frequencies for proxies of *CFH* Y402H, rs1410996, rs12144939 and rs10490924 were collected from previous investigations [34, 41]. Frequencies for *CFH* I62V could not be inferred from publicly available resources. Since effect sizes for rs12144939 and *CFH* I62V proxies were not available, we used a published and validated method [84, 85] to infer beta coefficients and standard errors (SE) from *p*-values for these two SNPs. Briefly, the method uses the fact that the sample size was the same for each variant to convert *p*-values to *z*-scores. Under the assumption that SE of the beta coefficient from a logistic regression is proportional to $1-\sqrt{\mathrm{MAF}(1-\mathrm{MAF})}$, where MAF is the minor allele frequency, then $\mathrm{SE}\times \sqrt{\mathrm{MAF}(1-\mathrm{MAF})}$ should be constant for all variants. We took the average of this term for the 34 genome-wide significant variants for which beta coefficients and SE were provided by the consortium (Table 1 of the published manuscript [34]). The SE for the remaining variants was then estimated by dividing the term by $\sqrt{\mathrm{MAF}(1-\mathrm{MAF})}$. The accuracy of this approach was validated using the 34 genome-wide significant variants [84].

## Supplementary Information

**Additional file 1.** Supplementary tables and figures.

## Data Availability

All relevant data and summary statistics are included in the manuscript or its supplementary materials. Access to genotype information is not provided to protect participants from identification. Additional data available upon request.
