# Supplementary material for: Protective chromosome 1q32 haplotypes mitigate risk for age-related macular degeneration associated with the CFH-CFHR5 and ARMS2/HTRA1 loci
Source: Hum Genomics. 2021 Sep 25;15:60. doi: 10.1186/s40246-021-00359-8 (PMC8466924; doi:10.1186/s40246-021-00359-8)
Supplement: Supplementary file 1 — Additional file 1. Supplementary tables and figures. [file 40246_2021_359_MOESM1_ESM.pdf]

Protective chromosome 1q32 haplotypes mitigate risk for age-related macular degeneration associated with the *CFH-CFHR5* and *ARMS2/HTRA1* loci

Chris M. Pappas[1], *Moussa A. Zouache*[1,*], *Stacie Matthews*[1], *Caitlin D. Faust*[1], *Jill L. Hageman*[1], *Brandi. L. Williams*[1], *Burt T. Richards*[1], *Gregory S. Hageman*[1,*]

[1]Steele Center for Translational Medicine, John A. Moran Eye Center, Department of Ophthalmology and Visual Sciences, University of Utah, Salt Lake City, UT 84132, United States

*Corresponding authors: moussa.zouache@hsc.utah.edu & gregory.hageman@hsc.utah.edu

Additional file 1

Figures

1. Figure S1: Odds ratios, counts and frequency among cases and controls for *CFH-CFHR5* and *ARMS2/HTRA1* diplotype combinations among cases and controls and Europeans from the 1000 Genomes Project (1000 G) phase 3.

2. Figure S2: Odds ratios and 95% confidence intervals generated separately for groups of individuals with either zero, one or two *ARMS2/HTRA1* risk alleles using Risk/Risk *CFH-CFHR5* diplotypes as a reference.

Tables

3. Table S1: Classification of stages of AMD employed.

4. Table S2: Haplotype analysis of the *CFH-CFHR5* extended region using *CFH* I62V, rs1410996, *CFH* Y402H and the *CFHR3/1* deletion-tagging SNP rs12144939.

6. Table S3: Haplotypes based on the protection-conferring *CFHR3/1* deletion, *CFH* I62V and the risk variant *CFH* Y402H with a frequency higher than 1% in the 1000 Genomes Project phase 3 (1000 G).

7. Table S4: *CFH-CFHR5* haplotype combinations (diplotypes) present with a frequency higher than 1% in our cohort and among Caucasians of the 1000 Genomes Project (1000 G) phase 3.

Figure S1: Odds ratios, counts and frequency among cases and controls for *CFH-CFHR5* and *ARMS2/HTRA1* diplotype combinations among cases and controls and Europeans from the 1000 Genomes Project (1000 G) phase 3. Effect sizes were calculated using Firth's bias-reduced logistic regression [57] while adjusting for age and gender. Bonferroni correction for multiple testing of 30 diplotype combinations = 0.0016 (0.05/30).

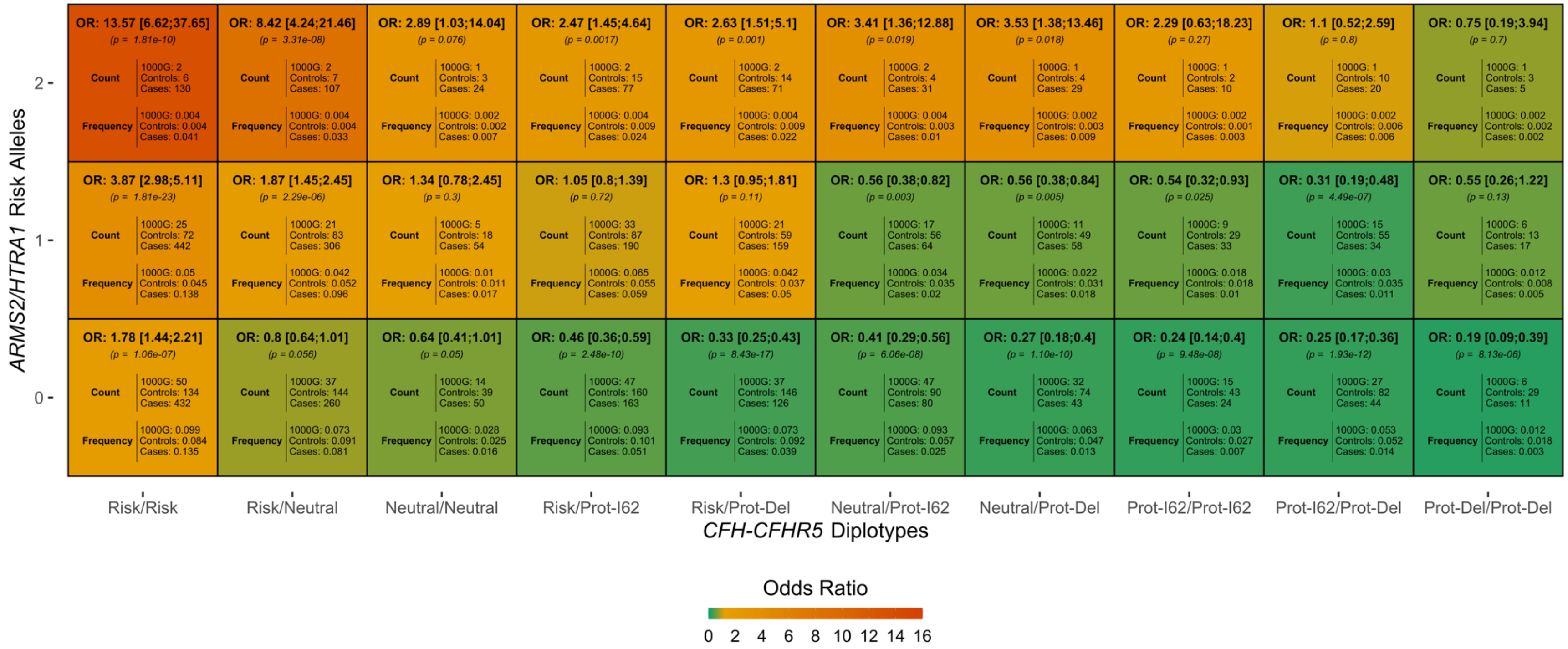

Figure S2: Odds ratios and 95% confidence intervals generated separately for groups of individuals with either zero, one or two *ARMS2/HTRA1* risk alleles using Risk/Risk *CFH-CFHR5* diplotypes as a reference. Bonferroni correction for multiple testing of 18 diplotype combinations = 0.0028 (0.05/18).

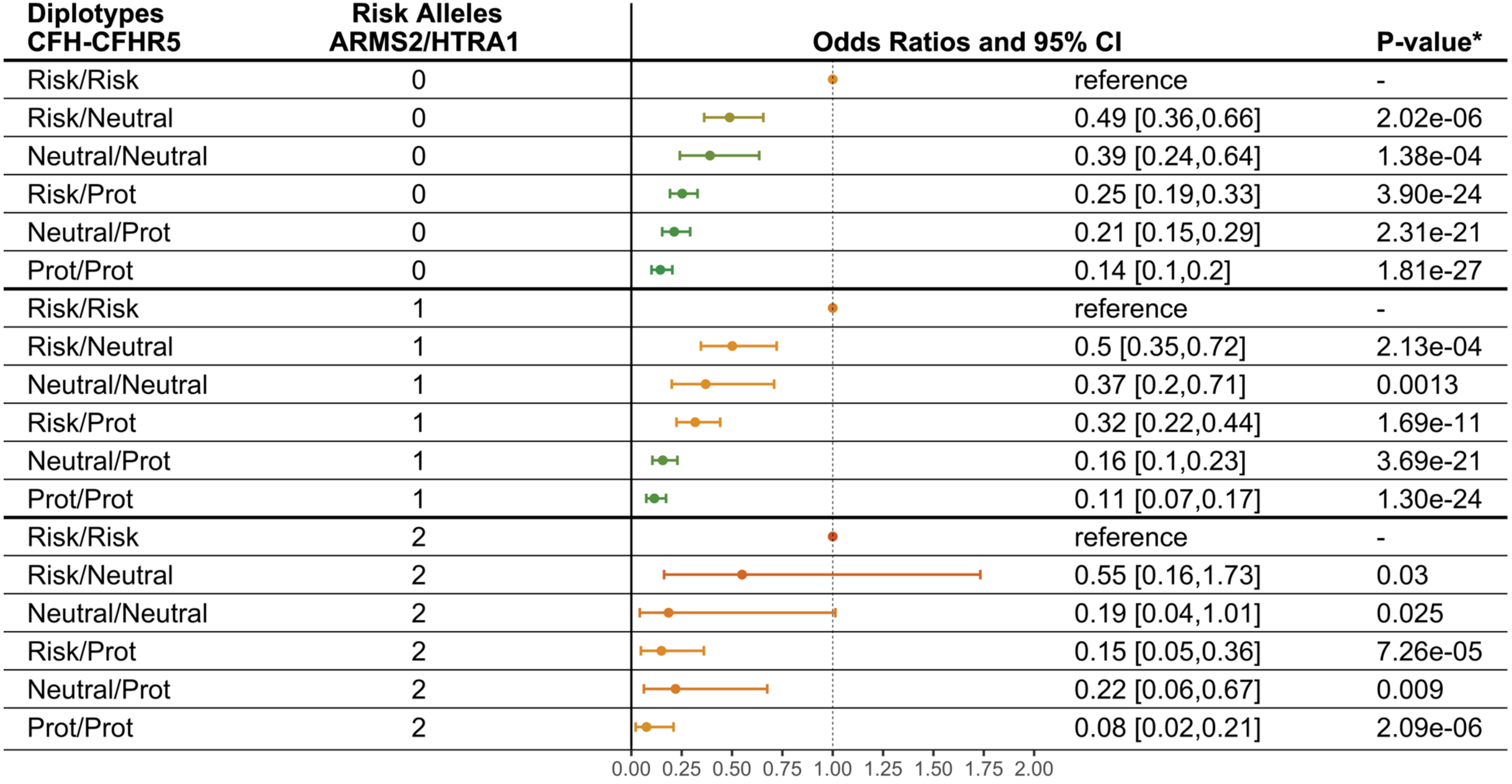

Diplotypes CFH-CFHR5	Risk Alleles ARMS2/HTRA1	Odds Ratios and 95% CI	P-value*
Risk/Risk	0	reference	-
Risk/Neutral	0	0.49 [0.36,0.66]	2.02e-06
Neutral/Neutral	0	0.39 [0.24,0.64]	1.38e-04
Risk/Prot	0	0.25 [0.19,0.33]	3.90e-24
Neutral/Prot	0	0.21 [0.15,0.29]	2.31e-21
Prot/Prot	0	0.14 [0.1,0.2]	1.81e-27
Risk/Risk	1	reference	-
Risk/Neutral	1	0.5 [0.35,0.72]	2.13e-04
Neutral/Neutral	1	0.37 [0.2,0.71]	0.0013
Risk/Prot	1	0.32 [0.22,0.44]	1.69e-11
Neutral/Prot	1	0.16 [0.1,0.23]	3.69e-21
Prot/Prot	1	0.11 [0.07,0.17]	1.30e-24
Risk/Risk	2	reference	-
Risk/Neutral	2	0.55 [0.16,1.73]	0.03
Neutral/Neutral	2	0.19 [0.04,1.01]	0.025
Risk/Prot	2	0.15 [0.05,0.36]	7.26e-05
Neutral/Prot	2	0.22 [0.06,0.67]	0.009
Prot/Prot	2	0.08 [0.02,0.21]	2.09e-06

Table S1: Classification of Stages of AMD Employed

Grade	Findings	Stage of AMD
**0**	No observable sign of AMD<br>Small hard drusen (< 63μm in diameter)	No AMD
**1A**	Soft distinct drusen (≥ 63μm in diameter)	Early AMD
**1B**	Isolated pigmentary changes only; no drusen (≥ 63μm in diameter)	
**2A**	Soft indistinct drusen only (≥ 125μm in diameter)	
**2B**	Soft distinct drusen (≥ 63μm in diameter) with pigmentary changes	
**3**	Soft indistinct drusen (≥ 125μm in diameter) with pigmentary changes	Intermediate AMD
**4A**	Presence of geographic atrophy	Late AMD
**4B**	Presence of neovascular membrane	
**4C**	Presence of neovascular membrane following geographic atrophy	

Table S2: Haplotype analysis of the *CFH-CFHR5* extended region using *CFH* I62V, rs1410996, *CFH* Y402H and the *CFHR3/1* deletion-tagging SNP rs12144939. Frequencies among Caucasians from the 1000 Genomes Project phase 3 (1000 G) are also provided.

Haplotype	*CFH* I62V (rs800292)	*CFH* Y402H (rs1061170)	rs1410996 (IAMGC Locus 1.1)	*CFHR3/1* Del (rs12144939)	Counts			Frequency			Score Statistic*	*p*-value**
	Prot: A	Risk: C	Prot: A	Del: T	1000 G	Controls	Cases	1000 G	Controls	Cases		
H1 (Risk)	G	C	G	G	356	1192	3599	0.354	0.364	0.555	17.53	8.97e-69
H2 (Protection)	A	T	A	G	229	656	791	0.228	0.210	0.125	-10.69	1.13e-26
H3	G	T	G	G	200	604	1217	0.199	0.196	0.195	-0.77	0.44
H4 (Protection)	G	T	A	T	177	606	659	0.176	0.192	0.104	-12.41	2.45e-35
H5 (Protection)	A	T	G	G	14	64	67	0.014	0.019	0.010	-3.34	8.5e-4
H6	A	T	A	T	11	23	20	0.011	0.008	0.004	-3.52	4.3e-4
H7	G	T	A	G	11	20	25	0.011	0.007	0.004	-1.64	0.10

*Based on the X^2-square test with one degree of freedom.
**Bonferroni correction for multiple testing of 7 haplotypes = 0.007 (0.05/7).

Table S3: Haplotypes based on the protection-conferring *CFHR3/1* deletion, *CFH* I62V and the risk variant *CFH* Y402H with a frequency higher than 1% in the 1000 Genomes Project phase 3 (1000 G). The common neutral haplotype H3, which describes the absence of genetic risk or protection, was used as the reference haplotype when generating odds ratios.

Haplotype	*CFH* I62V (rs800292)	*CFH* Y402H (rs1061170)	*CFHR3/1* Del (rs12144939)	Counts			Frequency			Score Statistic*	*p*-value**	OR (95% CI)	*p*-value**
	Prot: A	Risk: C	Del: T	1000 G	Controls	Cases	1000 G	Controls	Cases				
**H1 (Risk)**	G	C	G	356	1194	3601	0.354	0.364	0.555	17.51	1.22e-68	1.61 [1.42; 1.83]	1.22e-13
**H2 (Prot-I62)**	A	T	G	243	718	857	0.242	0.228	0.134	-11.34	7.72e-30	0.61 [0.52; 0.71]	1.9e-10
**H3 (Neutral)**	G	T	G	211	635	1245	0.210	0.204	0.200	-1.31	0.19	**1.0 (reference)**	-
**H4 (Prot-Del)**	G	T	T	177	596	655	0.176	0.191	0.104	-12.23	2.15e-34	0.53 [0.45; 0.62]	3.91e-14
**H5**	A	T	T	11	22	20	0.011	0.008	0.004	-4.01	6.12e-5	0.49 [0.23; 1.03]	0.059

*Based on the X^2-square test with one degree of freedom. **Bonferroni correction for multiple testing of 5 haplotypes = 0.01 (0.05/5)

Table S4: *CFH-CFHR5* haplotype combinations (diplotypes) present with a frequency higher than 1% in our cohort and among Caucasians of the 1000 Genomes Project (1000 G) phase 3. Effect sizes were calculated using the neutral diplotype (Neutral/Neutral) as a reference.

Diplotype	Overall Effect	Counts			Frequency			OR (95% CI)	*p*-value*
		1000 G	Controls	Cases	1000 G	Controls	Cases		
**Risk/Risk**	**Risk**	77	226	1053	0.147	0.145	0.333	2.56 [1.78; 3.64]	2.39e-7
**Risk/Neutral**	**Risk**	60	235	682	0.119	0.151	0.216	1.40 [0.97; 1.99]	0.07
**Neutral/Neutral**	**Neutral**	20	60	128	0.04	0.04	0.04	**1 (reference)**	-
**Risk/Prot-I62**	**Protection**	82	266	434	0.162	0.171	0.137	0.79 [0.55; 1.13]	0.20
**Risk/Prot-Del**	**Protection**	60	224	356	0.119	0.144	0.113	0.71 [0.49; 1.02]	0.06
**Neutral/Prot-I62**	**Protection**	66	151	176	0.131	0.097	0.056	0.56 [0.37; 0.83]	0.004
**Neutral/Prot-Del**	**Protection**	44	129	131	0.087	0.083	00.041	0.48 [0.32; 0.73]	5.73e-4
**Prot-I62/Prot-I62**	**Protection**	25	74	68	0.085	0.094	0.031	0.42 [0.26; 0.67]	3.38e-4
**Prot-I62/Prot-Del**	**Protection**	43	129	131	0.0495	0.048	0.022	0.48 [0.32; 0.73]	3.64e-7
**Prot-Del/Prot-Del**	**Protection**	13	45	33	0.0257	0.029	0.01	0.34 [0.19; 0.60]	2.45e-4

*Bonferroni correction for multiple testing of 10 diplotypes = 0.005 (0.05/10).
